# Assembly Patterns and Functional Roles of Bacterial Generalists and Specialists in the Remediation of Saline–Alkali Soils

**DOI:** 10.1111/1758-2229.70221

**Published:** 2025-10-26

**Authors:** Guanghao Wang, Keyu Yao, Jean Damascene Harindintwali, Eun Hea Jho, Jian Hu, Mingming Sun

**Affiliations:** ^1^ Soil Ecology Lab, Jiangsu Collaborative Innovation Center for Solid Organic Waste Resource Utilization and Jiangsu Key Laboratory for Solid Organic Waste Utilization Nanjing Agricultural University Nanjing China; ^2^ Engineering Research Center for Soil Nutrient Management and Pollution Remediation Institute of Soil Science, Chinese Academy of Sciences Nanjing China; ^3^ University of Chinese Academy Sciences Beijing China; ^4^ Department of Agricultural and Biological Chemistry Chonnam National University Gwangju Republic of Korea; ^5^ Jiangsu Geological Bureau Nanjing Jiangsu China; ^6^ Coastal Saline‐Alkali Land Ecological Rehabilitation and Sustainable Utilization Technology Innovation Center, MNR Nanjing Jiangsu China

**Keywords:** community assembly, generalists, saline alkaline land, specialists

## Abstract

Saline–alkali soils cover millions of hectares worldwide, severely limiting agricultural productivity. Although various remediation strategies have been applied, the adaptive responses of microbial communities to these interventions remain poorly understood. This study investigated microbial community responses to saline–alkali stress under different remediation treatments, focusing on diversity patterns, community assembly mechanisms, network interactions and functional roles. A randomised block experiment was conducted with three treatments: untreated saline–alkaline (SA) soils, paddy–upland rotation (PUR) and organic fertiliser (OF) amendment. Both PUR and OF treatments increased the relative abundances of *Bacteroidota*, *Acidobacteriota* and *Firmicutes*, indicating enrichment of beneficial taxa. Specialists exhibited higher connectivity with other microbial species than generalists, emphasising their role in stabilising indigenous microbiota and enhancing resistance to saline–alkali stress. Community assembly was dominated by deterministic processes for specialists in SA and PUR soils, while stochastic processes prevailed in other contexts across both generalists and specialists. These results reveal distinct but complementary roles of microbial generalists and specialists in soil adaptation and highlight the mechanisms by which amendments shape microbial community structure and function. Our findings provide mechanistic insights into microbial contributions to saline–alkali soil remediation, informing strategies for sustainable soil restoration.

## Graphical abstract

Microbial Community Response to Remediation in Saline‐Alkaline Soils.

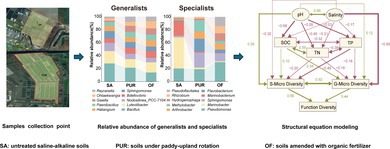

## 1 Introduction

Saline–alkaline soils are widespread, covering over 1.38 billion hectares globally (approximately 10.7% of the Earth's land surface) and pose a major threat to food security and soil ecosystem health (FAO 2024; Saifullah et al. 2018; Xiao et al. 2025; Xu et al. 2023). In China alone, these soils cover ~36.7 million hectares, of which ~12.3 million have agricultural potential and may be reclaimed, while the remainder is difficult to restore due to extreme salinity or high costs (Li et al. 2020; Wang et al. 2024). Saline–alkaline soils are characterised by high soluble salt concentrations, elevated pH values above 8.5, or both, which disrupt physical, chemical and biological soil properties (Shi et al. 2019; Zhang et al. 2022). Such conditions reduce water infiltration and aeration, alter nutrient cycling, inhibit microbial activity and limit crop growth, causing annual yield losses of 13%–58% (Fu et al. 2024; Haj‐Amor et al. 2022; Hassani et al. 2021; Zhang et al. 2024). Alarmingly, projections suggest that by 2050, nearly half of the world's arable land could be adversely affected by salinisation (Wang et al. 2020). Effective management of saline–alkaline (SA) soils is therefore critical to mitigate yield losses and ensure sustainable food production for a projected global population of 9.7 billion by 2050 (FAO 2020). Current remediation strategies predominantly rely on organic amendments and water management practices (Li et al. 2020; Zhang, Sun, et al. 2025; Zhang, Wu, et al. 2025). Organic fertilisers improve soil fertility by enhancing organic matter content, boosting nutrient availability and facilitating salt leaching (Wu et al. 2021; Xiao et al. 2025; Xie et al. 2020). Additionally, sustainable cropping systems, such as paddy–upland rotation (PUR), have shown promise in balancing desalination, water use efficiency and yield improvement, making them increasingly adopted interventions for SA soil management (Zhang, Sun, et al. 2025; Zhang, Wu, et al. 2025). The success of these remediation efforts depends critically on the soil microbial community, which plays a central role in nutrient cycling and ecosystem functioning.

Soil bacteria adapt to environmental filtering through diverse ecological niche strategies, broadly classified as generalists and specialists (Wang et al. 2025; Yang et al. 2023). Generalist bacteria possess broad environmental tolerance and maintain ecosystem functions across diverse habitats, thereby enhancing system resilience under changing conditions (Sriswasdi et al. 2017). In contrast, specialist bacteria thrive in narrow environmental niches and often exhibit strong functional traits that contribute to plant growth promotion and stress mitigation in specific conditions (Du et al. 2025; Rain‐Franco et al. 2022; Yan et al. 2022). These two groups form complementary guilds, stabilising community structure and function through ecological niche differentiation and cooperative interactions that buffer soil ecosystems against environmental perturbations (Xu et al. 2020). Understanding the assembly processes and ecological functions of microbial generalists and specialists is key to predicting soil microbial dynamics and designing microbiome‐informed remediation strategies.

Ecological community assembly theory provides a framework to understand how microbial communities respond to environmental stress (Wang et al. 2023). Community structure is shaped by deterministic processes, including environmental selection and biotic interactions and stochastic processes, such as ecological drift and dispersal limitation (Jiménez et al. 2023; Liu et al. 2023; Xu, Luo, et al. 2022). In SA soils, strong abiotic stress intensifies environmental filtering, producing distinct microbial structures and functions. Generalist bacteria exhibit broad environmental tolerance, with assembly largely governed by stochastic processes, stabilising community structure and supporting plant growth (Du et al. 2025; Niu et al. 2025; Schaerer et al. 2023). Specialists occupy narrow niches, and deterministic assembly enhances stress‐adaptive traits, including osmotic regulation and antimicrobial production, directly protecting plants (Li et al. 2021; Lindh et al. 2016). Together, these complementary guilds enhance plant tolerance and productivity under saline stress through cooperative interactions. Despite their key roles, most studies focus on overall diversity or functional gene abundance (Xu, Luo, et al. 2022; Yao et al. 2021), leaving the ecological and functional links between generalist and specialist abundance, assembly and plant performance underexplored. Elucidating these relationships is essential for predicting microbial contributions to soil resilience and designing microbiome‐informed strategies for sustainable crop production.

In this study, the ecological assembly and functional roles of generalist and specialist bacteria in SA soils were examined under different agricultural management regimes in coastal Jiangsu, China. High‐throughput 16S rRNA gene sequencing was used to profile bacterial communities across untreated and remediated soils. Integrating co‐occurrence network analysis, the normalised stochasticity ratio (NST) and neutral community model (NCM) revealed the ecological strategies and interaction patterns underlying microbial community assembly. This study focuses on (i) characterising the composition and traits of generalists and specialists under saline–alkali stress, (ii) elucidating their assembly mechanisms and network structures and (iii) assessing microbial functional stability and resistance to saline–alkali conditions. The results provide mechanistic insights into how microbial guilds adapt to environmental stress and offer guidance for microbiome‐based strategies to enhance soil restoration and sustainable agriculture in saline‐affected regions.

## 2 Materials and Methods

### 2.1 Site Description and Characteristics

The study was conducted in Nantong City, located in the southeastern part of Jiangsu Province, China (120°12′E–121°55′E, 31°41′N–32°43′N). The region exhibits a North Subtropical Monsoon climate, characterised by mild temperatures and distinct seasonal variations. The long‐term average annual temperature is approximately 15.1°C, with an annual precipitation of 1084 mm and annual evaporation of 857 mm (Cai et al. 2020). The terrain elevation generally ranges between 3.2 and 4.5 m above sea level. Two experimental sites were selected for this study: Tongzhou Bay (121°25′E, 32°15′N) and the Rudong Jueju Reclamation Zone (121°24′E, 32°16′N).

Both sites have been part of a long‐term land restoration programme initiated in 2010, focusing on soil amelioration, the development of salt‐tolerant crop varieties and the implementation of sustainable agricultural practices. In Tongzhou Bay, a rice–barley crop rotation system was adopted to facilitate seasonal alternation between flooding (for salt leaching) and dry cultivation (to enhance root development). This was combined with micro‐sprinkler irrigation systems to regulate soil moisture and salinity levels. In contrast, the Rudong Jueju Reclamation Zone employed bio‐organic fertiliser (OF) application (3 t ha^−1^) combined with deep tillage to promote soil desalination and fertility restoration.

### 2.2 Research Design and Procedures

Based on differences in land management practices, three representative treatments were selected for comparative analysis (Figure S1): untreated SA soil (control), soil under PUR and soil treated with OF. At each site, six soil cores (0–20 cm depth) were randomly collected in an ‘S’‐shaped sampling pattern across the plot and composited into a single representative sample (0–2.5 kg per plot). Soil samples were transported to the laboratory under cool conditions. Subsamples were stored at 4°C for DNA extraction and high‐throughput sequencing. The remaining portions were air‐dried, homogenised and sieved (< 0.25 mm) for physicochemical analyses.

### 2.3 Response Measurements

Basic soil properties, including salinity, soil organic carbon (SOC), soil pH, total nitrogen (TN) and total phosphorus (TP), were determined using standardised analytical protocols. Soil pH was measured in a 1:2.5 soil‐to‐water suspension using a glass electrode (Cheng et al. 2023). SOC content was quantified via the external heating potassium dichromate oxidation method (Cheng et al. 2022). TN was determined using the semi‐micro Kjeldahl digestion method (Mariano et al. 2017), and TP was measured using the NaOH extraction–molybdenum‐antimony colorimetric method (Qiao et al. 2018). All measurements were performed in triplicate and calibrated against certified reference materials to ensure analytical accuracy and data reliability.

### 2.4 Biomolecular Analyses

Total genomic DNA was extracted from 0.5 g of soil using E.Z.N.A. Soil/Stool DNA Kit (Omega Bio‐tek, Norcross, GA, USA) following the manufacturer's protocol. DNA purity and concentration were assessed using a NanoDrop One spectrophotometer (Thermo Fisher Scientific, USA). The V3–V4 region of the 16S rRNA gene was amplified by PCR using primers 515F/907R (515F: 5′‐GTGCCAGCMGCCGCGG‐3′ and 907R: 5′‐CCGTCAATTCMTTTRAGTTT‐3′) (Zhang, Sun, et al. 2025; Zhang, Wu, et al. 2025). The amplified PCR products were sent to Magigen Biotechnology Company (Guangzhou, China) for library construction and paired‐end sequencing (2 × 250 bp) on the Illumina MiSeq Platform. Raw fastq files were first demultiplexed using in‐house perl scripts according to the barcode sequences information for each sample with the following criteria: (i) The 250 bp reads were truncated at any site receiving an average quality score < 20 over a 10 bp sliding window, discarding the truncated reads that were shorter than 50 bp; (ii) exact barcode matching, two nucleotide mismatch in primer matching, and reads containing ambiguous characters were removed; and (iii) only sequences that overlap longer than 10 bp were assembled according to their overlap sequence. Reads that could not be assembled were discarded.

### 2.5 Microbial Analyses and Assessment

#### 2.5.1 Classification of Bacterial Generalists and Specialists

Operational taxonomic units (OTUs) were taxonomically classified by aligning representative sequences against the SILVA 16S rRNA reference database using the USEARCH algorithm (Quast et al. 2012). To distinguish bacterial generalists from specialists, we calculated the niche breadth (*H*_*i*_) of each OTU using Levin's niche breadth index:

$$H_{i}=1/\sum_{j=1}^{r}{\left(P_{\mathrm{ij}}\right)}^{2}$$

where *H*_*i*_ is the niche breadth of species *i*, *P*_*ij*_ is the proportional abundance of species *i* in location *j* and *r* is the total number of locations.

Niche breadth indices were computed using the EcolUtils package in R (Kokou et al. 2019) and compared against a null distribution generated by 1000 random permutations. Bacterial generalists were defined as OTUs with observed niche breadth values exceeding the upper 95% confidence interval (observed > uppCI), while specialists were identified as those below the lower 95% confidence interval (observed < lowCI) (Gao et al. 2023; Yan et al. 2022).

#### 2.5.2 Bacterial Community Assembly Analysis

To assess the relative contributions of deterministic and stochastic processes to bacterial community assembly, we employed the NST framework. NST quantifies the degree of stochasticity by comparing observed community dissimilarities with those expected under a null model that simulates random assembly (Ning et al. 2019). Higher NST values indicate a stronger influence of stochastic processes. Additionally, an NCM was applied to further evaluate the role of stochasticity in shaping indigenous bacterial communities. The model estimates the relationship between the occurrence frequency of taxa and their relative abundance in the metacommunity, under the assumption of ecological neutrality. Model fit was evaluated using the coefficient of determination (*R* ^2^), with higher *R* ^2^ values indicating a stronger contribution of stochastic processes to community assembly. The migration parameter *Nm*, defined as the product of the metacommunity size (*N*) and immigration rate (*m*), was used to represent the dispersal capacity of taxa across communities (Gao et al. 2023). Together, NST and NCM provide complementary insights into the balance between deterministic selection and stochastic dispersal in microbial community dynamics.

#### 2.5.3 Bacterial Functional Profiling

Bacterial functional potential was inferred using Phylogenetic Investigation of Communities by Reconstruction of Unobserved States (PICRUSt2) (Douglas et al. 2020). Briefly, representative 16S rRNA gene sequences were aligned with reference sequences to construct a phylogenetic tree. Using the Castor hidden‐state prediction algorithm, the gene family copy numbers were inferred by mapping the feature sequences onto the phylogeny based on their closest taxonomic relatives. The predicted copy numbers of gene families were then multiplied by the relative abundance of each feature in the corresponding sample to estimate functional profiles. Kyoto Encyclopedia of Genes and Genomes (KEGG) Orthologs (KOs), metabolic pathways and enzyme commission (EC) annotations were subsequently assigned. This enabled the generation of sample‐specific abundance tables of predicted gene families and functional categories at various hierarchical levels (Huang et al. 2021).

### 2.6 Data Analysis

Alpha diversity metrics for bacterial taxa and functional genes were calculated using the ‘diversity’ function in the vegan package (R v4.4.1). To evaluate differences in microbial community composition among treatments, principal coordinate analysis (PCoA) was performed based on Bray–Curtis dissimilarities, followed by analysis of similarity (ANOSIM) to test for statistically significant group differences (Yang et al. 2024). The top 30 most abundant generalists and specialists were selected for phylogenetic analysis. Representative sequences were aligned using MEGA 7.0, and phylogenetic trees were constructed using the neighbour‐joining method. Tree visualisations were refined and annotated using the Interactive Tree of Life (iTOL) platform (Yao et al. 2024). Microbial co‐occurrence network analysis was conducted in R to examine intra‐community correlations, generating edge and node files that were subsequently imported into Gephi (v0.9.2) for network visualisation following parameter optimisation (Zhang, Sun, et al. 2025; Zhang, Wu, et al. 2025). Given that niche breadth serves as an indicator of community adaptability and diversity patterns, niche breadth indices were calculated using the ‘niche. breadth’ function from the ‘spaa’ package in R. To evaluate community assembly processes, we employed the NST based on phylogenetic β‐diversity indices to quantify the relative contributions of deterministic versus stochastic factors (Sun et al. 2022). These results were further validated through NCM analysis implemented using the ‘hmisc’ and ‘NST’ packages in R. Structural equation modelling was performed with the ‘lavaan’ package. All statistical analyses were carried out using R v4.4.1. Data visualisation was accomplished using TBtools (v2.154), Origin 2024 and the ‘ggplot2’ package in R v4.4.1.

## 3 Results

### 3.1 Bacterial Community and Functional Composition in Saline–Alkaline Soils

#### 3.1.1 Generalist and Specialist Community Structures in Soils

**FIGURE 1:**
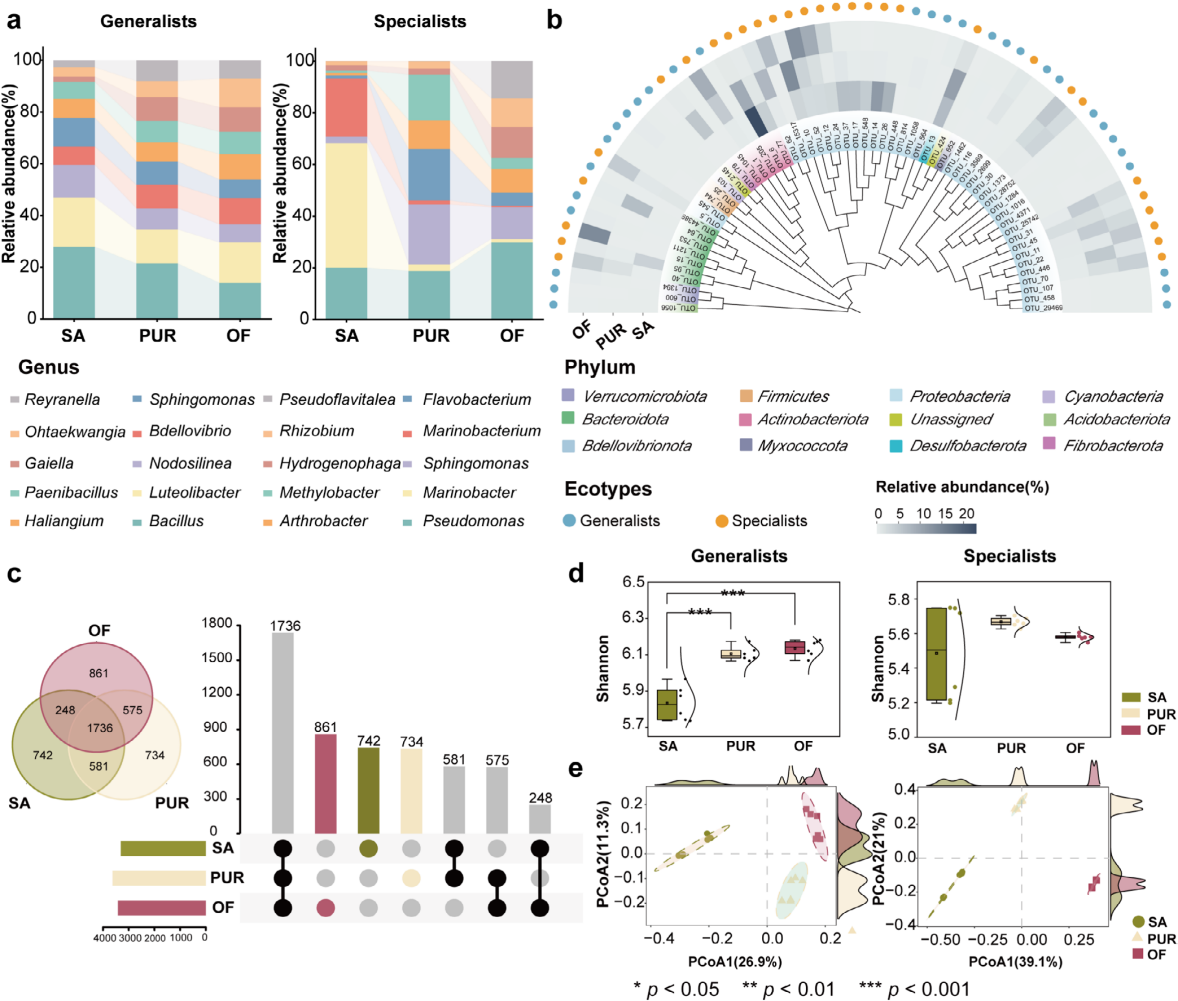
Bacterial community composition and diversity. Where SA is saline alkaline land, PUR is paddy–upland rotation, and OF is organic fertiliser. (a) Bacterial community composition at the genus level. The top 10 genera in relative abundance of generalists and specialists at the genus level across the three study sites. (b) The heatmap of phylogeny and bacterial species abundance of generalists and specialists across the three study sites. The innermost circle shows the phylogenetic tree of the different species, and the second circle shows the OTUs' names, where the background colours of these labels represent the relative abundance of corresponding species across all treatments. Meanwhile, the outermost circle shows the heat map and ecotype of the above species under different treatments, respectively. (c) Venn diagrams and codon usage plots of species composition across the three study sites. (d) Shannon indices of generalists and specialists in the soils of the three sample plots (the left panel shows the Shannon index of generalists, while the right panel displays that of specialists). (e) Beta diversity analysis of generalists and specialists in the soils of the three sample plots (the left panel displays the PCoA of generalist microbial communities, while the right panel shows that of specialist microbial communities).

The microbial species composition in the three sample plots of SA, PUR and OF is different, as shown in Figure S2. Compared to SA, the relative abundance of *Bacteroidetes*, *Acidobacteria* and *Firmicutes* in the soil of PUR and OF plots increased. Further analysis of the genus‐level species composition of generalists and specialists in the three plots revealed that the relative abundances of generalists and specialists varied significantly among treatments (Figure 1a). In SA, the dominant generalist genera were *Bacillus* (27.9%), *Luteolibacter* (19.0%) and *Nodosilinea* (12.6%), jointly comprising 59.5% of the total bacterial community. In PUR, the dominant generalists included *Bacillus* (21.5%), *Luteolibacter* (13.2%) and *Gaiella* (9.2%), accounting for 43.9% of the total bacterial community. In OF, *Luteolibacter* (15.7%), *Bacillus* (14.0%) and *Ohtaekwangia* (11.0%) were the most abundant generalists, representing 40.7% of the bacterial community. In contrast, specialist communities exhibited distinct dominant genera across treatments. In SA, specialists were dominated by *Marinobacter* (48.2%), *Marinobacterium* (22.5%) and *Pseudomonas* (20.1%), comprising 90.8% of the total bacterial community. In PUR, *Sphingomonas* (23.3%), *Flavobacterium* (19.9%) and *Pseudomonas* (18.8%) dominated, accounting for 62.0%. In OF, *Pseudomonas* (29.8%), *Pseudoflavitalea* (14.3%) and *Sphingomonas* (12.4%) were the most prevalent specialists, which together comprised 56.5% of the community.

A phylogenetic tree constructed from the top 30 generalist and specialist OTUs (ranked by relative abundance) across the three treatments revealed a dominance of *Proteobacteria* (36 OTUs) and *Bacteroidetes* (7 OTUs) (Figure 1b). Overall, specialists were more abundant than generalists across all soil groups. The most abundant generalist OTUs belonged to *Firmicutes*, *Proteobacteria* and *Bacteroidota*, while dominant specialist OTUs were affiliated with *Actinobacteria*, *Desulfobacterota* and *Proteobacteria*. Venn analysis of OTUs distributions (Figure 1c) showed that SA, PUR and OF contained 22.4%, 20.2% and 25.2% unique OTUs, respectively. A total of 1736 OTUs were shared among all three treatments. Notably, PUR and OF shared 575 OTUs, whereas OF and SA shared only 248, indicating a greater similarity between the managed soils. Shannon diversity indices (Figure 1d) for generalists followed the trend OF > PUR > SA (SA: 5.8; PUR: 6.1; OF: 6.2; *p <* 0.001), suggesting that organic fertilisation (OF) and crop rotation increased generalist diversity. In contrast, Shannon indices for specialists did not differ significantly among treatments. PCoA based on OTUs composition (Figure 1e) further confirmed significant differences in both generalist and specialist community structures across treatments (*p* < 0.05). Together, these findings indicate that different land management practices, particularly OF application and PUR, significantly altered the composition and diversity of soil bacterial communities. *Proteobacteria* emerged as the dominant phylum across all treatments, and improved soil conditions in OF and PUR contributed to enhanced microbial diversity.

#### 3.1.2 Ecological Functions of Soil Bacterial Communities

**FIGURE 2:**
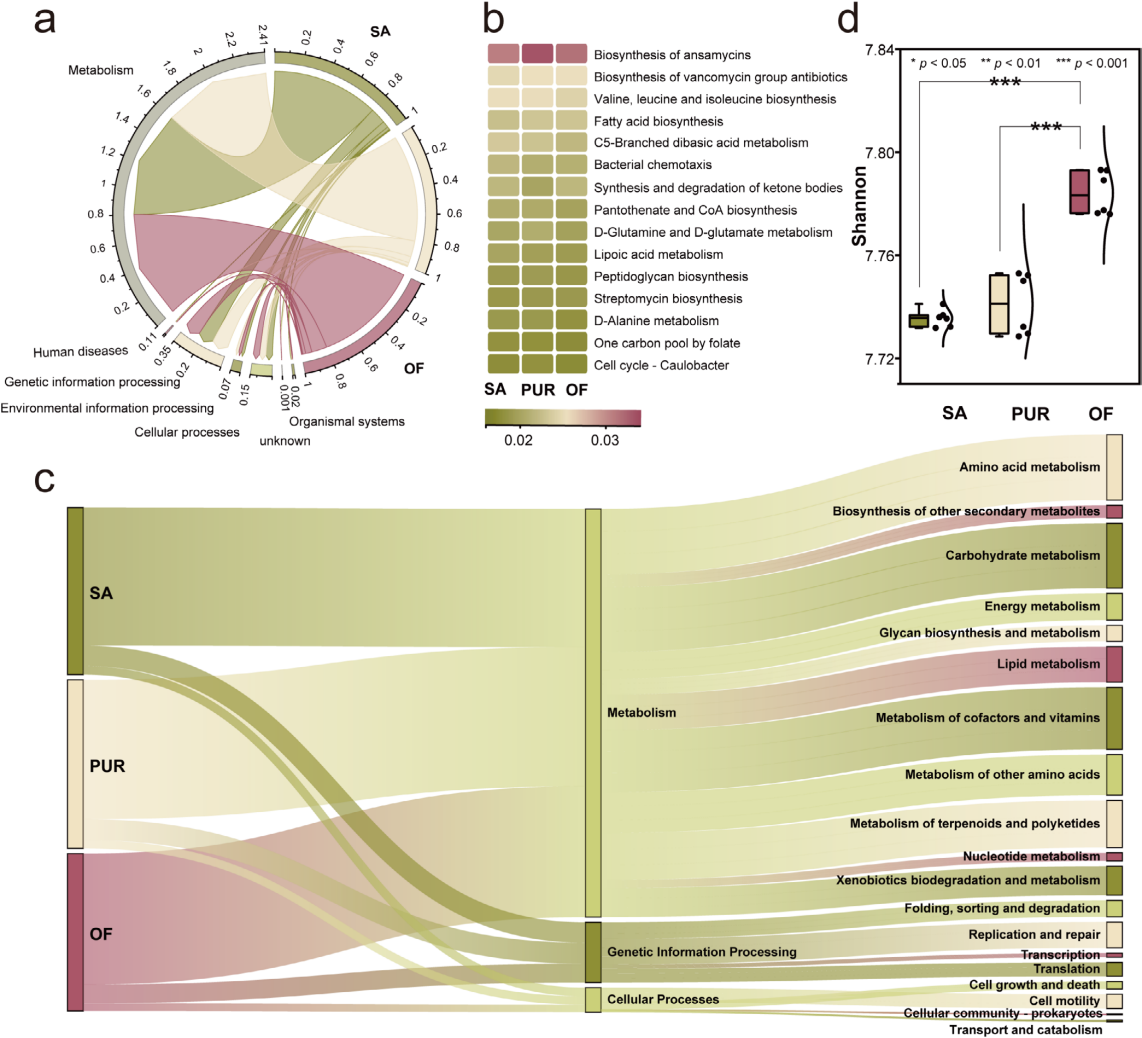
Analysis of KEGG metabolic pathways in bacterial communities. (a) Overview of the distribution of Level 1 functional metabolic genes in microbial communities across the three study sites. Among them, the green, yellow and red areas represent the distribution profiles of Level 1 functional metabolic genes in soil microbial communities from saline–alkali land (SA), paddy–upland rotation (PUR) and organic fertiliser (OF)‐amended sites, respectively. (b) Heatmap of relative abundance of Level 3 functional metabolic genes in microbial communities across the three study sites. (c) The relative abundance of the top three KEGG Level 1 pathways and their corresponding Level 2 metabolic pathways in soil microbial communities across the three study sites. Here, the green, yellow and red areas represent the KEGG Level 1 and Level 2 metabolic pathways in saline–alkali land, PUR and OF‐amended soils, respectively. (d) Bacterial gene diversity indices across the three study sites. Here, the green, yellow and red areas represent saline alkaline land, PUR and OF, respectively.

To investigate the functional differences in indigenous bacterial communities across treatments, metabolic pathways and potential ecological functions were predicted using KEGG‐based annotations. At KEGG Level 1, five broad metabolic categories were identified: cellular processes, environmental information processing, organismal systems, human diseases and genetic information processing. Compared to the control soil (SA), the relative abundance of cellular processes (SA, 5.0%; PUR, 4.8%; and OF, 4.7%) and environmental information processing (SA, 2.2%; PUR, 2.1%; and OF, 2.1%) decreased in improved soils (PUR and OF), while the relative abundance of organismal systems (SA: 0.5%, PUR, 0.6%; and OF, 0.6%) increased (Figure 2a). Further analysis of the top three most abundant KEGG Level 1 pathways and their corresponding Level 2 metabolic pathways revealed 19 secondary metabolic pathways, with amino acid metabolism (12.9%), carbohydrate metabolism (12.7%) and metabolism of cofactors and vitamins (12.2%) being the most dominant across all samples. Further analysis at KEGG Level 3 identified 188 pathways, of which the 15 most abundant were selected for detailed comparison (Figure 2b,c). Notably, pathways related to antibiotic biosynthesis were highly represented. The biosynthesis of ansamycins pathway exhibited the highest relative abundance (2.8%), followed by the biosynthesis of vancomycin group antibiotics (2.1%). The relative abundance of these pathways was consistently higher in improved soils compared to the control. Specifically, ansamycin biosynthesis accounted for 2.7% in SA, 2.9% in PUR and 2.8% in OF; vancomycin group antibiotic biosynthesis accounted for 2.0% in SA and 2.1% in both PUR and OF. These findings suggest that soil improvement practices, particularly PUR and OF treatments, enhance the metabolic potential of bacterial communities, especially in pathways related to secondary metabolite production. Additionally, microbial functional diversity, measured by the Shannon index, was significantly higher in the OF treatment compared to both PUR and SA (*p* < 0.001; Figure 2d). This indicates that the application of OF not only promotes specific functional pathways but also contributes to broader ecological functional diversity, thereby improving overall soil quality.

### 3.2 Adaptation of Bacterial Communities in Saline Alkaline Soils

#### 3.2.1 Co‐Occurrence Network Analysis

**FIGURE 3:**
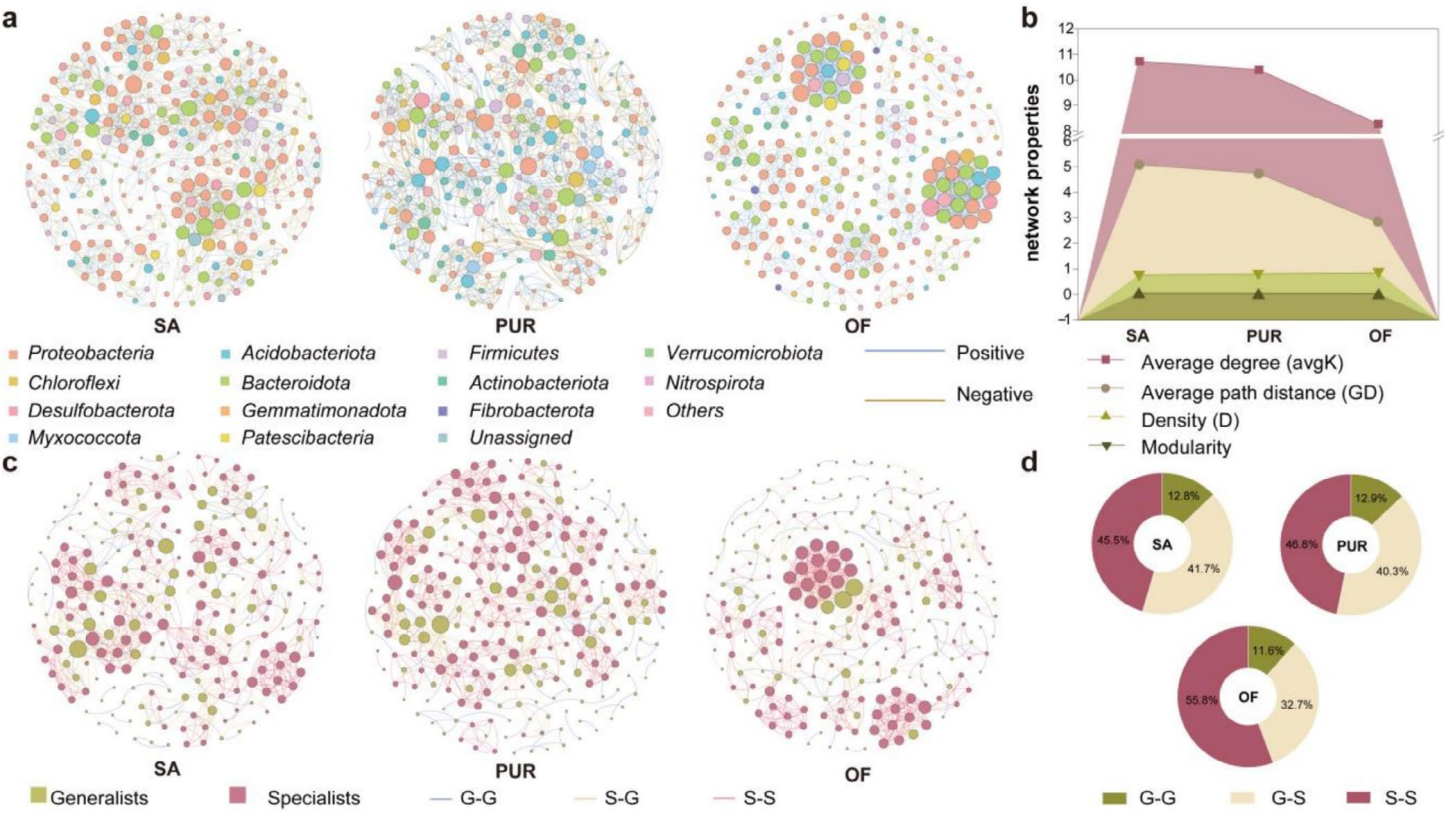
Association analysis among soil microorganisms in the three sample sites. (a) Co‐occurrence network diagram of the top 300 OTUs in relative abundance across the three study sites. Among them, different coloured circles indicate the classification of different species phyla, the size of the circles indicates the degree of association between species, dark blue lines indicate positive correlation between species, and yellow lines indicate negative correlation between species. (b) Network attributes of the co‐occurrence network diagrams of the top 300 OTUs in relative abundance across the three study sites, with red squares denoting average degree (avgK), green lower triangles denoting density (D), green circles denoting average path distance (GD) and dark green upper triangles denoting modularity. (c) Co‐occurrence network diagrams of the top 100 generalists and specialists in relative abundance in the soil of each of the three sample sites. The green circles indicate generalists, the red circles indicate specialists, Dark blue lines represent interactions between generalists only, yellow lines indicate interactions between generalists and specialists, and red lines denote interactions among specialists exclusively. (d) Proportions of edge types (generalist‐generalist, generalist‐specialist and specialist‐specialist) in soil microbial networks across the three study sites. Where green areas represent the proportion of generalist‐specialist edges, yellow areas indicate the proportion of generalist‐specialist edges; Red areas denote the proportion of specialist‐specialist edges.

To examine microbial interactions under different soil management regimes, co‐occurrence networks were constructed using the top 300 most abundant OTUs from the SA, PUR and OF treatments. The resulting microbial networks comprised 273 nodes and 1465 edges in SA, 268 nodes and 1394 edges in PUR, and 259 nodes and 1071 edges in OF. Improvement treatments significantly enhanced positive microbial associations. The proportion of positive correlations increased from 50.7% in SA to 57.3% in PUR and reached 87.6% in OF, indicating stronger mutualistic interactions among bacterial taxa under improved soil conditions (Figure 3a).

Network topology analysis revealed a progressive reduction in connectivity and complexity across treatments. The average degree (avgK) decreased from 10.73 in SA to 10.40 in PUR and 8.27 in OF, while network density (D) declined slightly (SA: 0.039; PUR: 0.038; and OF: 0.032). Notably, the average path length (GD) was shortest in OF (2.8), compared to PUR (4.7) and SA (5.1), suggesting tighter and more efficient microbial interactions in the organically amended soil. Modularity, a measure of network compartmentalisation, increased with soil improvement (SA: 0.76; PUR: 0.80; and OF: 0.84), indicating enhanced ecological niche differentiation (Figure 3b).

To further resolve microbial interaction patterns, the co‐occurrence networks of the top 150 most abundant generalist and specialist taxa were analysed. Across all treatments, interactions between specialists (S–S) dominated the networks, increasing from 45.5% in SA to 46.8% in PUR and 55.8% in OF. Specialist–generalist (S–G) interactions were intermediate (SA: 41.7%; PUR: 40.3%; and OF: 32.7%), while generalist–generalist (G–G) interactions were consistently the lowest (SA: 12.8%; PUR: 12.9%; and OF: 11.6%) (Figure 3c,d). These findings suggest that soil improvement practices enhance network stability by strengthening interactions among specialists, which may contribute to increased microbial niche specialisation and community resilience.

#### 3.2.2 Assembly Processes of Soil Bacterial Communities

**FIGURE 4:**
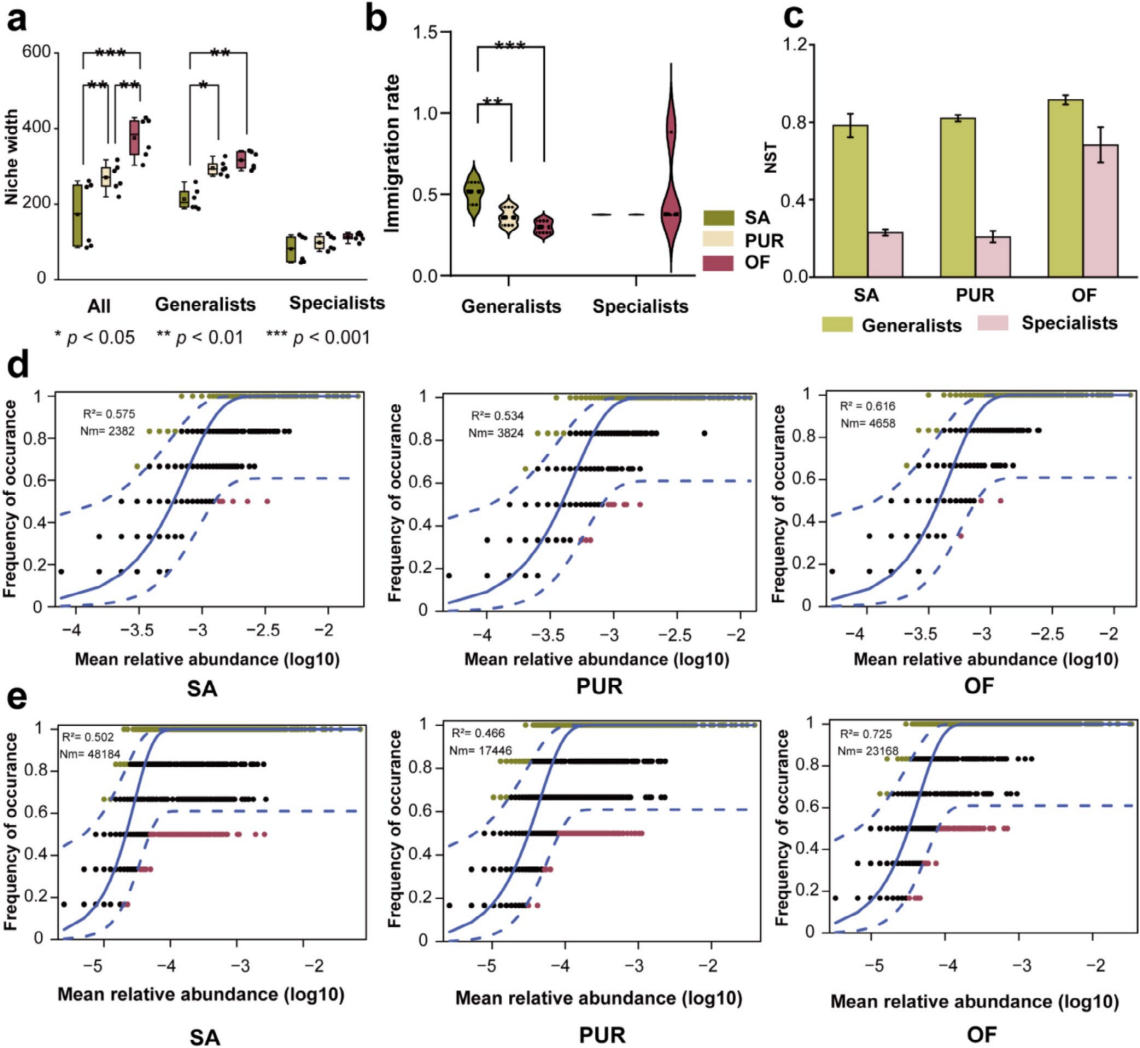
Assembly mechanisms of native bacterial communities. (a) Ecological niche widths of all species, generalists and specialists across the three study sites. Where green, yellow and red represent the saline–alkaline land, paddy–upland rotation and organic fertiliser‐amended plots, respectively. (b) Migration rates of generalist communities and specialist communities across the three study sites. Where green, yellow and red represent the saline–alkaline land, paddy–upland rotation and organic fertiliser‐amended plots, respectively. (c) NST values of generalist communities and specialist communities across the three study sites. The light green colour indicates the generalists community, and the pink colour indicates the specialists community. (d) Neutral community model for generalists across different sample plots. (e) Neutral community model for specialists across different sample plots.

To evaluate the mechanisms shaping bacterial community structure across treatments, dispersal limitation was quantified by calculating the average migration rate of bacterial taxa, and ecological assembly processes were assessed using null models. Following the OF amendment, the ecological niche breadth of both total bacterial communities and generalist taxa increased significantly (*p* < 0.01; Figure 4a), suggesting broader environmental adaptability. Migration rate analysis revealed that the dispersal rate of generalist taxa was highest in the untreated control (SA: 0.51), but decreased significantly in improved soils (PUR: 0.38; OF: 0.30; *p* < 0.01; Figure 4b). In contrast, the migration rates of specialist taxa remained relatively stable across all treatments (*p* > 0.05), indicating a more constrained dispersal capacity.

To further examine community assembly dynamics, the NST was calculated based on a null model, revealing that values for generalists exceeded 0.5 in all treatments (Figure 4c), indicating that their community assembly was consistently dominated by stochastic processes. In contrast, the NST values for specialists were below 0.5 in SA and PUR, reflecting deterministic assembly processes, but exceeded 0.5 in OF, suggesting a shift towards stochasticity following organic amendment. These findings were corroborated by the NCM, which describes the relationship between OTU frequency and relative abundance (Figure 4d,e). A higher *R* ^2^ value indicates a greater influence of stochastic processes. The generalist community exhibited moderate fits to the neutral model (SA: *R* ^2^ = 0.575; OF: *R* ^2^ = 0.616), while the specialist community showed a notable increase in model fit in OF (SA: *R* ^2^ = 0.502; OF: *R* ^2^ = 0.725), indicating that OF application reduced the strength of environmental filtering and increased the role of stochasticity in community assembly. Together, these results suggest that soil improvement, particularly through organic amendments, alters microbial assembly processes by expanding ecological niche breadth, limiting dispersal of generalists and weakening deterministic environmental filters, thereby restructuring microbial community dynamics in SA soils.

### 3.3 Key Environmental Drivers of Bacterial Community Structure in Saline–Alkaline Soils

**FIGURE 5:**
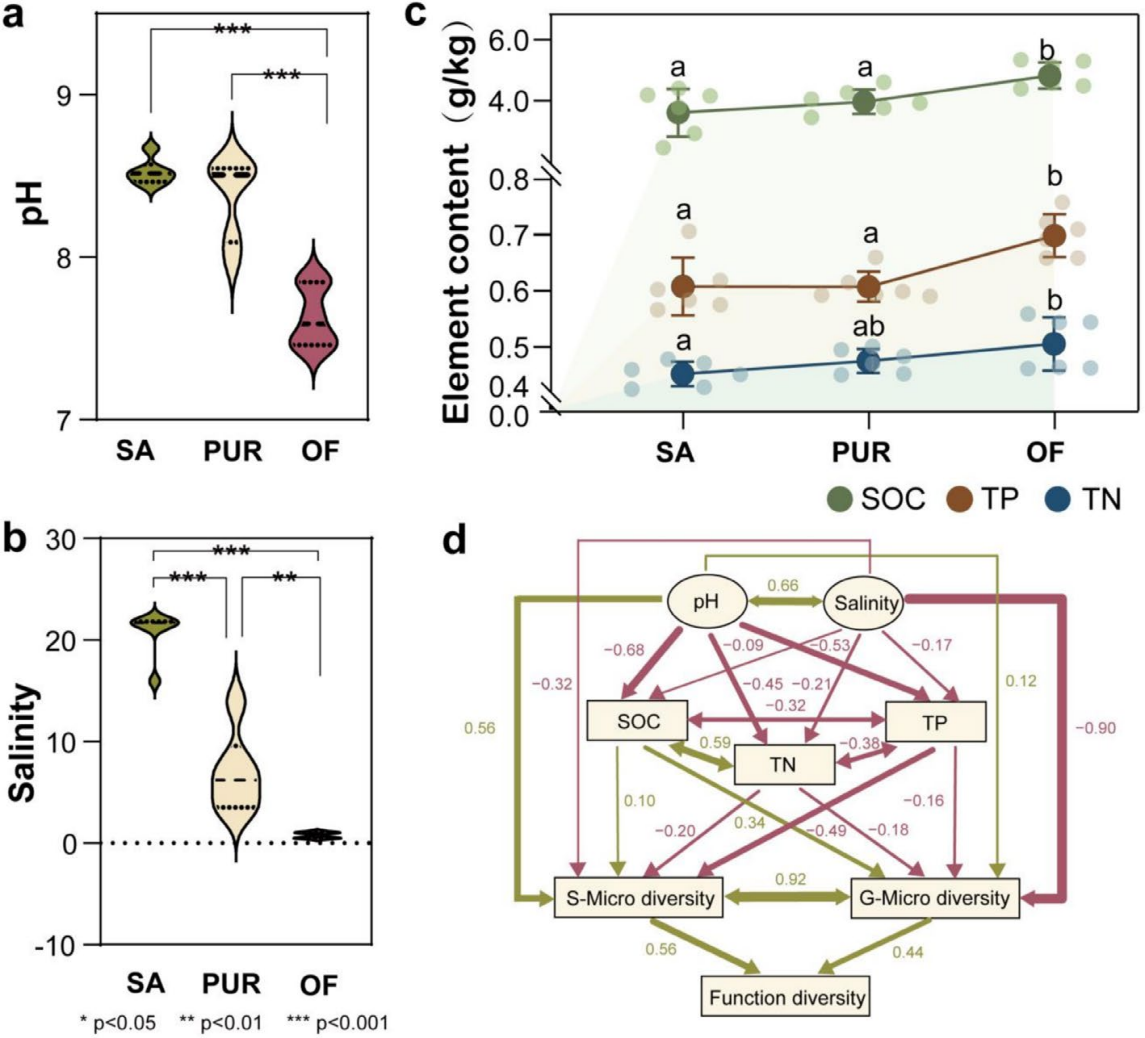
Effects of environmental factors on generalist diversity, specialist diversity and functional diversity. (a) Soil pH values across the three sample plots. Where green, yellow and red represent the saline–alkaline land, paddy–upland rotation and organic fertiliser, respectively. (b) Salinity across the three sample plots. Where green, yellow and red represent the saline–alkaline land, paddy–upland rotation and organic fertiliser, respectively. (c) Soil nutrient content across the three sample plots. Among them, green, red and blue circles represent SOC, TP and TN contents in soil, respectively. (d) Structural equation modelling between soil nutrients, generalists and specialists diversity, functional diversity and salinity. Where green lines indicate positive correlations, while red lines represent negative correlations.

To identify the major environmental variables shaping bacterial community composition across treatments, random forest analysis was employed. Among the tested factors, soil salinity and pH emerged as the dominant drivers of community differentiation (Figure S3). To further assess their impact, salinity and pH levels were compared across SA, PUR and OF sampling sites. Both PUR and OF treatments significantly reduced soil salinity and pH compared to the untreated control (*p* < 0.05), with the greatest reductions observed in the OF treatment (Figure 5a,b). In addition to mitigating salinity and alkalinity, both treatments improved soil fertility parameters. Levels of SOC, TN and TP followed the order OF > PUR > SA, with all increases statistically significant (*p* < 0.05; Figure 5c). These findings indicate that both PUR and OF application are effective land management strategies for alleviating SA stress and enhancing soil fertility. To further explore the relationships among soil properties, microbial community composition and functional attributes, a structural equation model (SEM) was constructed (Figure 5d). The SEM revealed that salinity negatively affected microbial functional diversity by suppressing the biodiversity of both generalist and specialist taxa. In contrast, pH exerted a positive influence on microbial biodiversity, indirectly enhancing functional diversity. These results highlight the central role of soil physicochemical properties, particularly salinity and pH, in regulating microbial structure and ecosystem function in SA environments.

## 4 Discussion

### 4.1 Improvement Measures Drive Microbial Community Structural Transformation and Functional Enhancement

Understanding how microbial communities respond to environmental improvement is essential for evaluating and enhancing soil fertility under changing conditions. In this study, we systematically explored the effects of SA land remediation on microbial community structure, functional capacity and ecological interactions, focusing on generalist and specialist taxa across untreated (SA) and treated (PUR and OF) soils. Soil improvement practices, particularly PUR and OF application, led to marked shifts in bacterial community composition. Notably, the relative abundances of *Bacteroidota*, *Acidobacteriota* and *Firmicutes* increased in improved soils (Figure S2). These taxa are functionally important: *Bacteroidota* and *Acidobacteriota* are key decomposers involved in organic matter turnover and nutrient cycling (Huang et al. 2015), while Firmicutes are known for their resilience to environmental stress (Huang et al. 2023). The enrichment of these groups suggests enhanced microbial functions that support soil health and plant productivity under reduced SA stress. This study found significant differences in bacterial community structure between PUR and OF application treatments, which reflects the distinct impacts of these two agricultural management practices on microbial ecological strategies. The relative abundance of the *Bacteroidetes* was significantly higher under OF treatment (22.54%) than under PUR treatment (14.03%). *Bacteroidetes* are a key functional group involved in degrading soil organic matter, particularly complex macromolecules such as proteins (Solden et al. 2017). The application of OF enhanced the availability of soil nutrients (e.g., organic carbon, nitrogen and phosphorus), providing ample metabolic substrates that stimulated the growth of *Bacteroidetes*. Consistent with previous studies, organic amendment promotes the proliferation of organic matter‐decomposing microorganisms like *Bacteroidetes* by improving soil nutrient status and supplying labile organic carbon (Dincă et al. 2022).

### 4.2 Soil Improvement Reshapes Microbial Functional Metabolism and Interaction Networks

Probiotic taxa, including specific members of the genera *Bacillus* and *Flavobacterium*, were notably enriched among generalists, whereas salt‐tolerant genera such as *Marinobacterium* and *Marinobacter* were predominantly associated with specialist communities (Figure 1a,b) (Dong et al. 2023; Eichorst et al. 2018; Huang et al. 2020; Liu et al. 2024). This indicates that soil amendments not only shift community composition but also enhance the functional potential of microbiota by selecting for taxa with beneficial or stress‐resistant traits. Despite these changes, the diversity of specialists remained stable across treatments, whereas generalists exhibited significantly higher diversity in improved soils (Figure 1c,d). This likely reflects the enhanced nutrient availability and reduced salinity, which provide a more favourable environment for broadly adapted taxa (Roberts and Stewart 2018), while specialists, which rely on specific niches, respond less dynamically (Rain‐Franco et al. 2022). KEGG pathway analysis revealed that soil improvement enhanced microbial metabolic functions associated with ecosystem health. Specifically, the relative abundance of pathways related to the biosynthesis of ansamycins and vancomycin‐group antibiotics increased in PUR and OF treatments, while pathways associated with C5‐branched dibasic acid metabolism declined (Figure 2a,b). Since the C5‐branched dibasic acid pathway is associated with halophytic stress responses (Nie et al. 2023), its reduction suggests a mitigation of salt stress. Conversely, the enrichment of antibiotic biosynthesis pathways may reflect increased microbial competition and ecological complexity, which contribute to soil resilience. These compounds are also known to suppress soil pathogens and support beneficial microbial taxa (Wu et al. 2023; Yao et al. 2024). Organic fertiliser application significantly enhanced the functional diversity (Shannon index) of the microbial community compared to both PUR and SA (*p* < 0.001; Figure 2d), indicating that nutrient enrichment promotes a broader array of microbial functions and improves soil quality. Microbial co‐occurrence network analysis revealed that improved soils harboured more positively correlated bacterial communities (Figure 3a,b), suggesting strengthened synergistic interactions and enhanced network stability. These changes likely result from the transition of microbial interaction structures, from competition‐dominated in SA soils to cooperation‐dominated in amended soils. This shift facilitates resource sharing and functional complementarity, contributing to a more stable and resilient microbial ecosystem. Interestingly, interaction networks revealed that specialists maintained strong intra‐group connectivity (Figure 3c,d), reinforcing their role in preserving core community functions under stress. These taxa serve as ecological anchors, providing resistance to environmental fluctuations and supporting community integrity.

### 4.3 Assembly Mechanisms of Generalist and Specialist Microbial Communities and Their Linkages to Plant Interactions Under Saline–Alkaline Stress

Niche breadth analysis demonstrated that OF significantly expanded the ecological range of both total bacterial communities and generalists (Figure 4a). The species migration rate of generalists was highest in untreated soils (SA), suggesting that environmental stress favours stochastic dispersal strategies. Assembly pattern analysis confirmed that generalists were predominantly structured by stochastic processes across treatments, while specialists, particularly in SA and PUR, were shaped by deterministic processes (Figure 4c–e). This is consistent with the results of previous studies (Liao et al. 2016; Xu, Luo, et al. 2022).

Previous studies indicate that stochastic processes lead to higher diversity and transition rates, suggesting that generalists dominated by stochastic processes are more critical for maintaining community functional stability (Xu, Vandenkoornhuyse, et al. 2022). Compared to generalists dominated by random processes, specialists dominated by deterministic processes exhibit enhanced functionality, thus contributing more to ecosystem function in specific environments (Graham and Stegen 2017). In saline–alkali environments, the assembly of microbial communities exhibits a close ecological relationship with plant tolerance to environmental stress. Generalists, shaped by stochastic processes, maintain the stability of fundamental ecological functions through extensive resource utilisation, providing sustained nutritional support to plants. In contrast, specialists, shaped by deterministic processes, enhance plants' precise adaptability by efficiently performing specific functions. Under OF treatment, organic amendments promote the stochastic assembly of specialists. Together with generalists, they form a more resilient microbial network. Through functional complementarity and niche differentiation, this significantly enhances plant survival in saline–alkali environments. This functional complementarity mechanism is further corroborated by existing research: under saline–alkali stress, both generalist and specialist microbial communities possess distinct yet indispensable functions, jointly exerting beneficial effects on plants. Generalist microbial communities predominantly promote plant growth, while specialist communities demonstrate varying protective potentials under different stresses through mechanisms such as osmoregulation and antibiotic secretion (Liu et al. 2025).

**FIGURE 6:**
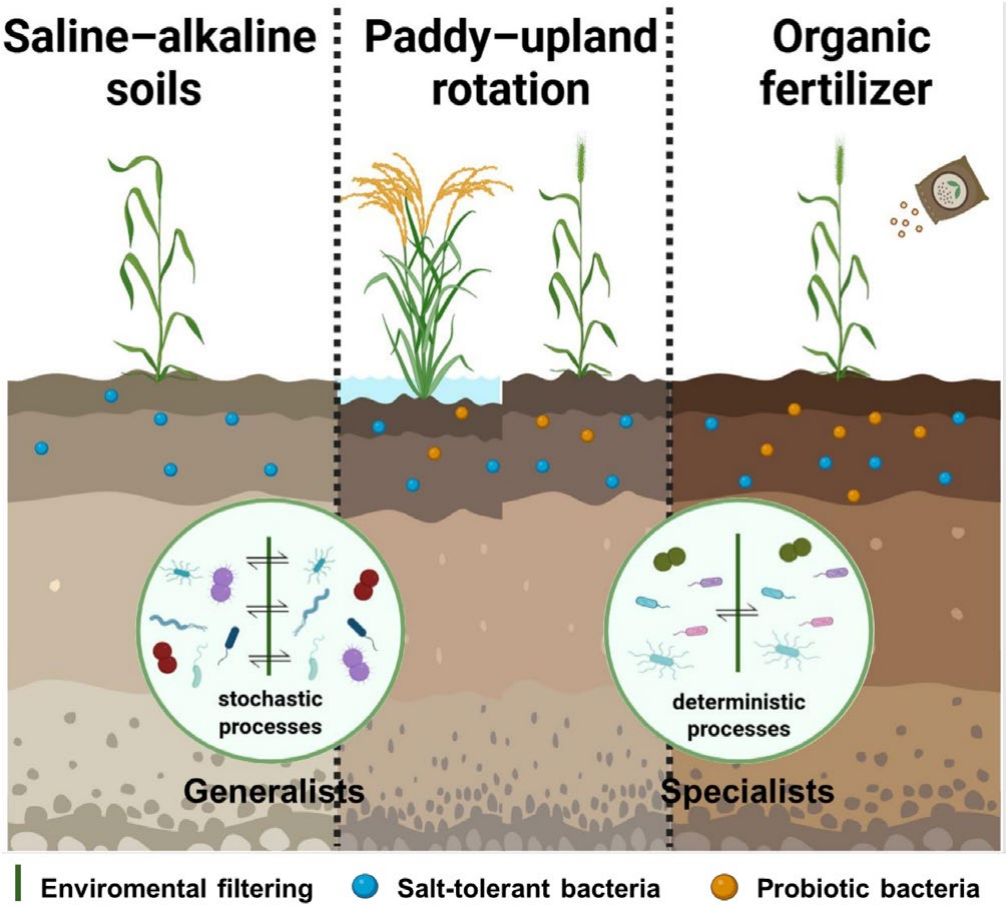
Microbial ecological strategies in saline–alkaline environments.

Structural equation modelling further confirmed that the diversity of both generalists and specialists significantly influenced overall functional diversity in SA soils (Figure 5), underscoring their complementary ecological roles. Generalists and specialists fulfil distinct but synergistic ecological functions (Wang et al. 2025). Generalists contribute to core metabolic functions and community diversity, while specialists perform niche‐specific roles such as ion regulation and stress adaptation (Cao et al. 2014). The coexistence and interaction of these groups foster ecological balance, enhance ecosystem stability and accelerate recovery in degraded environments. Together, these findings reveal that tailored soil improvement strategies, particularly those enhancing nutrient inputs and reducing salt stress, can restructure microbial communities, enrich functional potential and promote a shift from stress‐tolerant to health‐promoting soil ecosystems (Figure 6).

## 5 Conclusions

This study reveals how microbial functional adaptation underpins the ecological improvement of SA soils. Soil amelioration practices, whether PUR or OF amendment, led to significant shifts in bacterial community composition and structure, enhancing both functional capacity and environmental resilience. Indigenous microbial communities contributed not only to sustaining SA resistance but also to promoting beneficial, probiotic‐like traits within the soil ecosystem. Our findings underscore the complementary roles of generalists and specialists in community assembly. Specialists were key to maintaining community stability and stress resistance in high‐salinity conditions, whereas generalists exhibited greater metabolic flexibility, enabling functional diversification and ecosystem adaptation under improved conditions. By integrating community assembly theory with functional predictions, this work provides mechanistic insights into microbial ecological strategies in SA environments. These insights offer a scientific basis for optimising microbial communities to maintain stable, resilient and functionally diverse soils under salinity stress.

## Author Contributions

**Guanghao Wang:** writing – original draft, visualisation, formal analysis. **Keyu Yao:** investigation, methodology. **Jean Damascene Harindintwali:** visualisation, investigation. **Eun Hea Jho:** investigation. **Jian Hu:** resources, conceptualisation. **Mingming Sun:** writing – review and editing, validation, supervision, project administration, data curation.

## Conflicts of Interest

The authors declare no conflicts of interest.

## Supporting information


**Figure S1:** Map of sampling site locations. Among them, the upper right corner is the Rudong Jueju Reclamation Zone saline–alkali soil improvement area, and the lower right corner is the Tongzhou Bay saline–alkali soil improvement area.
**Figure S2:** The relative abundance of the top 10 phyla of all species at the phylum level.
**Figure S3:** Feature importance ranking derived from random forest model.

## Data Availability

The data that support the findings of this study are available on request from the corresponding author. The data are not publicly available due to privacy or ethical restrictions.
