# Supplementary material for: Assembly Patterns and Functional Roles of Bacterial Generalists and Specialists in the Remediation of Saline–Alkali Soils
Source: Environ Microbiol Rep. 2025 Oct 26;17(5):e70221. doi: 10.1111/1758-2229.70221 (PMC12554386; doi:10.1111/1758-2229.70221)
Supplement: Supplementary file 1 — Figure S1: Map of sampling site locations. Among them, the upper right corner is the Rudong Jueju Reclamation Zone saline–alkali soil improvement area, and the lower right corner is the Tongzhou Bay saline–alkali soil improvement area. Figure S2: The relative abundance of the top 10 phyla of all species at the phylum level. Figure S3: Feature importance ranking derived from random forest model. [file EMI4-17-e70221-s001.docx]

 **Fig. S1. Map of sampling site locations.** Among them, the upper right corner is the Rudong Jueju Reclamation Zone saline-alkali soil improvement area, and the lower right corner is the Tongzhou Bay saline-alkali soil improvement area.

**Fig. S2. The relative abundance of the top ten phyla of all species at the phylum level.**

**Fig. S3. Feature importance ranking derived from random forest model.**
